# Fbxo16 mediates degradation of NF-κB p65 subunit and inhibits inflammatory response in dendritic cells

**DOI:** 10.3389/fimmu.2025.1524110

**Published:** 2025-06-03

**Authors:** Akiko Sugimoto-Ishige, Aya Jodo, Takashi Tanaka

**Affiliations:** Laboratory for Inflammatory Regulation, RIKEN Center for Integrative Medical Sciences, Yokohama, Kanagawa, Japan

**Keywords:** F-box protein, NF-κB, ubiquitin E3 ligase, inflammation, PDLIM2

## Abstract

Activation of transcription factor NF-κB is tightly regulated by negative regulatory systems that prevent excessive inflammation leading to autoimmune diseases. We previously demonstrated that PDLIM2, a PDZ-LIM domain-containing nuclear protein, functions as a ubiquitin E3 ligase that targets the p65 subunit of NF-κB and STAT3/STAT4 transcription factors for proteasomal degradation, thus terminating immune responses in dendritic cells and CD4^+^T cells, respectively. In this study, we have demonstrated that PDLIM2 forms a ubiquitin ligase complex with Cullin 1, a scaffold protein, providing a platform consisting of complex and Skp1, an adaptor protein. Moreover, by screening using siRNA for F-box-containing proteins, we have identified Fbxo16 as a substrate-recognition receptor for p65 in this complex. Fbxo16 bound to p65 and promoted its polyubiquitination and degradation, thereby suppressing NF-κB transactivation. Consistently, Fbxo16 deficiency in dendritic cells resulted in a larger amount of nuclear p65 and thus enhanced production of proinflammatory cytokines. On the other hand, Fbxo16 could not promote the degradation of STAT3 or STAT4, and Fbxo16 deficiency did not affect STAT3- and STAT4-mediated immune responses of CD4^+^T cells. These results delineate a role of Fbxo16, as a substrate receptor for p65 in a PDLIM2-containing ubiquitin ligase complex, in negatively regulating NF-κB-mediated inflammatory responses in dendritic cells.

## 1 Introduction

Dendritic cells detect invading pathogens by their sensors, such as Toll-like receptors (TLR). TLR stimulation activates dendritic cells to induce inflammatory responses through the activation of the nuclear factor κB (NF-κB) transcription factor. In unstimulated cells, the p65/p50 heterodimer of NF-κB is associated with IκBα and sequestrated in the cytoplasm. TLR signaling results in proteasomal degradation of IκBα. NF-κB p65/p50 then enters the nucleus and induces the expressions of various inflammation-related genes, such as interleukin-6 (IL-6) and IL-12 (1). IL-6 and IL-12 then activate signal transducer and activator of transcription 4 (STAT4) and STAT3 in naïve T-helper (Th) cells and direct the differentiation of these cells into distinct Th subsets, Th1 and Th17 cells, respectively. Th1 and Th17 cells produce effector cytokines, such as IFN-γ and IL-17 respectively and fight against different types of microbial pathogens (2). On the other hand, these immune responses should be tightly regulated by negative regulatory systems to prevent excessive inflammation leading to the onset of autoimmune diseases or allergic diseases (3, 4). Notably, genome-wide association studies (GWAS) have demonstrated that variants of negative regulators of these signaling pathways, including A20, CYLD and IRAK-M, are associated with human immune disorders, including inflammatory bowel diseases, rheumatoid arthritis, and asthma (5–7).

PDLIM2 (also designated as SLIM or mystique), that belongs to a LIM protein family, is a nuclear protein containing both PDZ (designated after the first-identified three proteins; PSD-95, Dig and ZO-1) and LIM (designated after the first-identified three proteins; Lin-11,Isl-1 and Mec-3) domains (8–10). We have previously demonstrated that the LIM domain possesses ubiquitin E3 ligase activity (9) and PDLIM2 functions as a nuclear ubiquitin E3 ligase for the p65 subunit of NF-κB in dendritic cells (11). PDLIM2 not only promotes polyubiquitination of p65 through its LIM domain, but also shuttles p65 to the intranuclear compartments, PML (promyelocytic leukemia protein) nuclear bodies, where p65 is finally degraded by the proteasome, thereby negatively regulating NF-κB activity. Consistently, PDLIM2 deficiency resulted in enhanced production of proinflammatory cytokines in dendritic cells. In addition, PDLIM2 also promotes polyubiquitination and degradation of STAT4 and STAT3 transcription factors in CD4^+^T cells and inhibits the differentiation of naïve T cells into Th1 and Th17 cells (9, 12). Thus, PDLIM2 negatively regulates immune responses in both innate and acquired immunity, preventing autoimmune diseases (13).

We have recently focused on the analysis of how PDLIM2-mediated NF-κB p65 degradation in the nucleus is controlled in dendritic cells. We have previously demonstrated that the chaperone protein HSP70 binds to PDLIM2 in the nucleus and promotes the shuttling of NF-κB to the proteasome together with the proteasome-associated protein BAG-1 (14). We have further shown that PDLIM7, another LIM protein, forms heterodimer with PDLIM2 and promotes its K63-linked ubiquitination and then facilitate the transport of the NF-κB-PDLIM2 complex to the proteasome together with the ubiquitin-recognizing protein p62/Sqstm1 (15). However, the detailed molecular mechanisms of how PDLIM2 recognizes and polyubiquitinates target proteins remain unclear.

LIM domain is structurally related to the RING (really interesting new gene) finger domain, that has ubiquitin E3 ligase activity (16). Both LIM domain and RING finger domains consist of eight conserved cysteines (Cys) or histidine (His) residues, so the LIM domain might be a subtype of RING finger domain (17). To date, over 600 RING finger proteins are identified as ubiquitin E3 ligases. RING-type ubiquitin ligases are mainly classified into two types, which are monomer-type and multi-subunit complex-type (18). The multi-subunit complex-type ubiquitin E3 ligase is composed of one core scaffold protein Cullin, providing a platform of the complex, thus this complex is called the Cullin-Ring ubiquitin ligase complex (CRL) (19, 20). Cullin has seven family members, including Cullin 1, 2, 3, 4A, 4B, 5 and 7. One end of each Cullin protein binds to a substrate-recognizing receptor directly or through an adaptor protein. For example, Cullin 3 directly binds to BTB domain-containing substrate receptors, whereas Cullin 1 associates with various substrate receptor F-box proteins through an adaptor Skp1. The other end of Cullin binds to RING finger protein, an E2 enzyme-recruiting subunit, which facilitates ubiquitin transfer onto the substrate bound to the substrate receptor (21, 22). In this study, we have demonstrated that PDLIM2 forms a functional ubiquitin E3 ligase complex CRL1 with Cullin 1 and Skp1 together with Fbxo16 as a substrate-recognition component. Fbxo16 recognizes p65 and promotes its polyubiquitination and degradation cooperatively with PDLIM2, thereby suppressing p65-mediated NF-κB transactivation. In contrast, Fbxo16 did not polyubiquitinate and promote the degradation of STAT4 or STAT3, other substrates of PDLIM2 for ubiquitin-dependent degradation (9, 12). Consistently, Fbxo16 deficiency in dendritic cells resulted in augmented production of p65-mediated proinflammatory cytokines, whereas STAT4 and STAT3-mediated Th1 and Th17 differentiation of CD4+T cells were not affected by Fbxo16 deficiency. These results demonstrated that Fbxo16 is a p65-recognizing component in the PDLIM2-containing ubiquitin E3 ligase complex and inhibits NF-κB-dependent inflammatory responses in dendritic cells.

## 2 Materials and methods

### 2.1 Expression plasmids

For the c-Myc-tagged Fbxo16, the complementary DNA (cDNA) of mouse *Fbxo16* (GeneBank accession: NM_001114088) was amplified by KOD-Plus-Neo DNA polymerase (TOYOBO) and subcloned into pCMV-Myc (Clontech). For the c-Myc-tagged ΔF-box mutant of Fbxo16 (ΔF), the coding region between amino acids 92–129 was deleted from the full-length c-Myc-Fbxo16 plasmid by mutagenesis with In-Fusion cloning using primers; 5’-CTACCAAGATGCTCAAGTGCCTGCGC-3’ and 5’-TGAGCATCTTGGTAGTAAAATCCAG-3’. The c-Myc-tagged PDLIM2, Flag-tagged p65 and Flag-p65-IRES-Venus were previously described (9, 11). For the HA-tagged PDLIM2, a HA-tag plus the coding sequence of murine *Pdlim2* (GeneBank accession: NM_145978) was inserted into pCMV- Myc (Clontech), as a result, the c-Myc-tag was replaced with the HA-tag. For the Flag-tagged PDLIM2, mouse *Pdlim2* was subcloned into pCMV-DYKDDDDK (Clontech). Expression plasmids for Flag-tagged murine p50 (#20018), murine TRAF6 (#21624), murine MyD88 (#13093) and human IKKβ (#23298) were purchased from Addgene. The pRL-Null renilla construct (#E2271) was purchased from Promega. The ELAM-1 luciferase reporter construct was provided by D. Golenbock (23).

### 2.2 Reagents and antibodies

Lipopolysaccharide (LPS; from *Salmonella typhimurium*; L-2262) was purchased from Sigma-Aldrich/Merck. CpG oligonucleotides (ODN 1668, #tlrl-1668) were purchased from InvivoGen. MG132 (#474791) was purchased from Calbiochem/Merck. Murine GM-CSF (#415-ML) was purchased from R&D Systems. Human ligand for the receptor tyrosine kinase Flt3 (Flt3L) (#300-19) was purchased from Peprotech. Anti-DYKDDDDK (NU01102) antibody, that corresponds to an anti-Flag antibody, was purchased from Nacalai USA. Anti-p65 (sc-372), p50 (sc-7178), IκBα (sc-371) and PKC (sc-10800) antibodies were from Santa Cruz Biotechnology. Anti-LSD1 (#2184), cdc37 (#3604), Histone H3 (#4499) Lamin B1 (#12586) and Skp1 (#12248) antibodies were from Cell Signaling Technology. Anti-HSP90 (#13171-1-AP) and Cullin 7 (#13738-1-AP) antibodies were from Proteintech. Anti-Cullin 1 (ab75817), Cullin 2 (ab166917) and Cullin 3 (ab75851) antibodies were from Abcam. Anti-γ1-actin (#016-27821) antibody were purchased from FUJIFILM Wako Pure Chemicals. HRP-conjugated anti-c-Myc antibody (M047-7), anti-c-Myc antibody-conjugated agarose (M047-8), anti-HA antibody (M180-3), and DDDDK-tagged Protein PURIFICATION GEL (#3328R) (used for immunoprecipitation of Flag-tagged proteins) were purchased from MBL. Anti-ubiquitin antibody (clone FK-2; BML-PW8810) was from Enzo Life Sciences. As a secondary antibody, HRP-goat anti-rabbit IgG (#111-035-003) was purchased from Jackson ImmunoResearch and HRP-conjugated sheep anti-mouse IgG (NA931) was purchased from GE Healthcare.

### 2.3 Cell culture, transfection, and reporter assay

Mouse embryonic fibroblasts (MEFs) were prepared as previously described (15). GM-CSF-differentiated bone marrow cells (GM-CSF-BMCs), Flt3L-differentiated bone marrow-derived dendritic cells (BMDC), macrophages, CD4^+^T, CD8^+^T and CD19^+^B cells were prepared as previously described (15). Human embryonic kidney (HEK) 293T cells, MEFs, GM-CSF-BMCs and BMDC were cultured in DMEM supplemented with 10% FCS. CD4^+^T cells were cultured in RPMI1640 supplemented with 10% FCS.

Effectene transfection reagents (QIAGEN) was used for transient transfection. For the reporter assay with the Dual Luciferase Reporter System (Promega), MEFs and HEK293T cells were transfected with the ELAM−1 luciferase and pRL-Null renilla reporter constructs and analyzed as previously described (15). Luciferase activity of the ELAM−1 luciferase reporter was normalized to the renilla luciferase activity and represented as fold-change to the control.

### 2.4 Subcellular fractionation, immunoprecipitation, and ubiquitination assay

All lysis buffers contained a protease inhibitor (Roche). Cytoplasmic, nuclear soluble and insoluble fractions were extracted as previously described (11). Anti-cdc37 (cytoplasm), anti-LSD1 (nuclear soluble fraction), and anti-Lamin B or Histone H3 (nuclear insoluble fraction) were used for checking the purity of the fractionation. For co-immunoprecipitation between Skp1 and PDLIM2 or Fbxo16, cells were lysed in 50 mM Tris pH 8.0, 0.5% NP-40, 5 mM EDTA, 50 mM NaCl, 50 mM sodium fluoride. For other co-immunoprecipitation experiments, cells were lysed in RIPA buffer (25 mM Tris pH 8.0, 150 mM NaCl, 1% NP-40, 1% sodium deoxycholate, 0.1% SDS). Whole cell extracts were then incubated with anti-c-Myc agarose or DDDDK-tagged Protein PURIFICATION GEL (MBL) overnight and washed four times with lysis buffer and analyzed by immunoblot analysis as previously described (15). For ubiquitination assay, His-tagged proteins were purified from whole cell lysates with His60 Nickel Superflow Resin (TAKARA BIO INC.) as previously described (24) and analyzed by immunoblot analysis.

### 2.5 Immunofluorescence staining

HEK293T cells, cultured on poly-L-lysine-coated slides, were transfected with Flag-p65-IRES-Venus without or with wild-type or ΔF mutant of Fbxo16, and analyzed by immunofluorescence staining as previously described (11).

### 2.6 Knockdown experiments with siRNA

HEK293T cells were first transfected with siRNA by Lipofectamine RNAiMAX (Thermo Fisher Scientific Inc.), then transfected with indicated plasmids and lysed with RIPA buffer and subjected to immunoprecipitation or immunoblot analysis. GM-CSF-BMCs were transfected with siRNA by the Neon Transfection System (Invitrogen) and analyzed as previously described (15). The siRNAs for 39 human Fbxo proteins (**Supplementary Table 1**), 10 murine Fbxo proteins (**Supplementary Table 3**) and control siRNA (12935–300) were selected from Stealth Select RNAi predesigned siRNAs (Thermo Fisher Scientific Inc.) and purchased.

### 2.7 Real-time PCR analysis

The synthesis of cDNA and the quantitative real-time PCR analyses were performed as previously described (15). To analyze the expression of proinflammatory cytokines in GM-CSF-BMCs, we used Taqman Fast Advanced Master Mix and probes for mouse *Il-6* (Mm00446190), *Il-12b* (Mm00434174), *Il-1β(Mm00434228), Cxcl2* (Mm00436450), *Cxcl10* (Mm00445235), *Csf3* (Mm00438334) and 18S rRNA (4319413E) from the TaqMan Gene Expression Assay (Applied Biosystems/Thermo Fisher Scientific). To analyze the expression of human and murine FBXO proteins, we used Fast SYBR Green Master Mix (Applied Biosystems/Thermo Fisher Scientific), and the primer pairs shown in **Supplementary Tables 2** ; **Supplementary Table 4** (Thermo Fisher Scientific Inc.). The cycle threshold (Ct) value of each gene expression was normalized to that of 18S rRNA and the fold-change to the control was calculated by the ΔΔCt method.

### 2.8 Generation of *Fbxo16*-deficient mice

The murine genomic *Fbxo16* allele was knocked out using the Alt-R CRISPR-Cas9 system (IDT) with the target sequence, TTTTACTACCAAGCTTCCAA(GGG). The crRNA containing this target sequence and tracrRNA were synthesized by IDT, mixed with Cas9 protein forming an RNP complex, and microinjected into pronuclei of C57BL/6J embryos. Mice were screened by PCR and sequencing analysis to determine the mutated region. The mutant line, in which deletion of a part of exon IV and intron IV in genomic DNA introduces a premature termination codon at the beginning of exon V, was selected for further study. This mutation deleted almost the entire region of the F-box domain, which is essential for Fbxo16 activity (**Supplementary Figure 1**). The mice with this deletion were crossed with wild-type C57BL/6J mice, that were purchased from CREA Japan, Inc, to generate heterozygous mice, and homozygotes were obtained by crossing of heterozygotes. The sequences of primer sets used for genotyping were as follows. Forward: 5’-GACTCTCTTGTTCCCTGTTTCCTC-3’ and Reverse: 5’-AGGTGGCAATGCTGTCCTACTGAG-3’. Mice were kept under specific pathogen free conditions and used for the experiments between 4 and 5 weeks of age. All experiments were approved by the Institutional Animal Care and Use Committee (IACUC) of RIKEN Yokohama Branch and performed in accordance with the committee’s guidelines.

### 2.9 T-helper cell differentiation experiments

CD4^+^ T cells were purified from spleen by MACS and cultured with plate-bound anti-CD3 (0.1 µg/ml) plus soluble anti-CD28 (1 µg/ml) for 3 days under Th1 and Th17 subset skewing conditions as follows; IL-12 (1 ng/ml) for Th1; TGF-β (0.5 ng/ml) plus IL-6 (10 ng/ml) for Th17, and then restimulated with plate-bound anti-CD3 (1 µg/ml) for 20 h and the production of IFN-γ and IL-17A in the supernatants was measured by ELISA (BD Biosciences).

### 2.10 Statistical Analyses

The student’s t test was used for all the statistical analysis. Data are represented as the mean values ± the standard deviation of the mean (SD).

## 3 Results

### 3.1 PDLIM2 bound to Cullin 1 and Skp1 forming a ubiquitin E3 ligase complex

**Figure 1:**
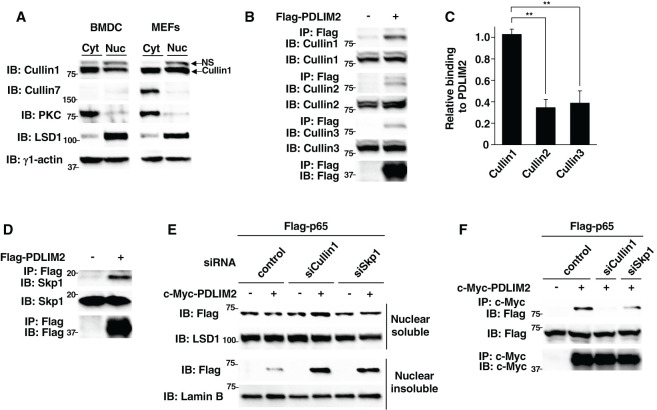
PDLIM2 bound to Cullin 1 and Skp1 forming a ubiquitin E3 ligase complex. **(A)** Expression of Cullin 1 and Cullin 7 in cytoplasmic and nuclear extracts of dendritic cells (BMDC) and fibroblasts (MEFs). The purity of the fractions of cytoplasmic and nuclear extracts was checked with anti-PKC and anti-LSD1 antibodies, respectively. NS, Non-specific band. **(B)** Interaction of PDLIM2 and Cullins in whole cell lysates of HEK293T cells, transfected with Flag-PDLIM2, then immunoprecipitated (IP) with anti-Flag, and immunoblotted (IB) with anti-Cullin 1, Cullin 2 or Cullin 3. **(C)** Densitometric analysis of the coimmunoprecipitated amount of each Cullin protein relative to the total amount of input. Shown are the mean ± SD. **P < 0.01. **(D)** Interaction of PDLIM2 and Skp1 in whole cell lysates of HEK293T cells, transfected with Flag-PDLIM2, then immunoprecipitated with anti-Flag, and immunoblotted with anti-Skp1. **(E)** Effect of Cullin 1 or Skp1 deficiency on PDLIM2-mediated p65 degradation in soluble and insoluble nuclear extracts of HEK293T cells, first transfected with control siRNA or siRNA against Cullin 1 or Skp1, then transfected with Flag-p65 without or with c-Myc-PDLIM2 and analyzed by immunoblot with anti-Flag antibody. **(F)** Effect of Cullin 1 or Skp1 deficiency on the interaction of PDLIM2 and p65 in HEK293T cells, first transfected with control siRNA or siRNA against Cullin 1 or Skp1 and then transfected with Flag-p65 without or with c-Myc-PDLIM2. Whole cell lysates were immunoprecipitated with anti-c-Myc and immunoblotted with anti-Flag. Data are representative of three **(A-F)** independent experiments.

During our initial study using mass-spectrometry-based proteomic screening for proteins coimmunoprecipitated with PDLIM2, we found that Skp1 is one of the PDLIM2-interacting proteins. Since Skp1 is an adaptor protein in the Cullin 1 and Cullin 7-containing Cullin-RING-ligase complexes (CRL1 and CRL7, respectively) (19), we predicted that PDLIM2 could form a complex with Cullin 1 and/or Cullin 7 plus Skp1 to function as a ubiquitin E3 ligase. We first clarified the subcellular localization of Cullin 1 and Cullin 7 in dendritic cells (BMDC) and mouse embryonic fibroblasts (MEFs) by immunoblot analysis (**Figure 1A**). The ratios of nuclear/cytoplasmic expression of Cullin 1 or Cullin 7 in each blot were calculated using densitometric analysis (**Supplementary Figure 2**). Cullin 1 was found in both the cytoplasm and nucleus in dendritic cells and MEFs. On the other hand, Cullin 7 expression was detected only in the cytoplasm in MEFs and was not detected in dendritic cells. The purity of the cytoplasmic and nuclear fractions was demonstrated by anti-PKC and LSD1 antibodies, respectively. These data suggest that Cullin 1, but not Cullin 7, could contribute to the PDLIM2-dependent degradation of nuclear p65 in dendritic cells. We therefore focused on Cullin 1 for the following studies.

We then determined if PDLIM2 can bind to Cullin 1. HEK293T cells, transfected with Flag-tagged PDLIM2, were immunoprecipitated with an anti-Flag antibody and then immunoblotted with anti-Cullin 1 and also anti-Cullin 2 and Cullin 3 as controls. Although PDLIM2 could bind to all the Cullin proteins we tested (**Figure 1B**), we demonstrated that PDLIM2 more strongly associated with Cullin 1 compared to Cullin 2 and Cullin 3 by densitometric analysis of the coimmunoprecipitated amount of each Cullin relative to the total amount of input (**Figure 1C**). Moreover, consistent with our previous study described above, PDLIM2 could also bind to Skp1 in this assay (**Figure 1D**). We previously demonstrated that PDLIM2 polyubiquitinated p65 and then transported p65 to the insoluble nuclear compartments, where p65 was degraded by the proteasome (11). To examine the effect of Cullin 1 and Skp1 on p65 degradation by PDLIM2, we knocked down Cullin 1 or Skp1 in HEK293T cells transfected with p65 without or with PDLIM2 and examined the levels of soluble and insoluble nuclear p65 protein. Knockdown of either Cullin 1 or Skp1 resulted in the increase of p65, mostly in the insoluble nuclear fraction, where p65 is degraded (**Figure 1E**), suggesting that both Cullin 1 and Skp1 are required for p65 degradation by PDLIM2. Moreover, the binding between PDLIM2 and p65 was impaired by the specific knockdown of either Cullin 1 or Skp1 (**Figure 1F**, **Supplementary Figure 3**). These data demonstrate that PDLIM2 can bind to Cullin 1 and Skp1 forming a CRL1 complex to function as a ubiquitin E3 ligase mediating p65 degradation.

### 3.2 Fbxo16 is a substrate-recognizing component in the PDLIM2-containing ubiquitin E3 ligase complex

In the PDLIM2-containing CRL1 complex, PDLIM2 is thought to be an E2-recruiting subunit and the substrate receptor should be some F-box protein (17–19). This idea prompted us to identify the F-box protein that functions as a substrate receptor for p65 and promotes its degradation in the nucleus. More than sixty F-box proteins have been identified thus far in humans and mice. These F-box proteins are classified into three classes depending on their domain structure in the molecule, namely the FBXW family (containing the F-box and a WD40 domain), the FBXL family (containing the F-box and a Leu-rich repeat) and the FBXO family which has only the F-box domain (21, 22). To date, the functions of these F-box proteins have been intensively studied mainly in cell cycle regulation and tumorigenesis. In contrast, the roles of F-box proteins in immune regulation remain unclear. Since the FBXO family is the largest subfamily of F-box proteins, we focused on it in our attempt to identify the FBXO protein involved in the binding to p65 in the PDLIM2-containing CRL1 complex.

**Figure 2:**
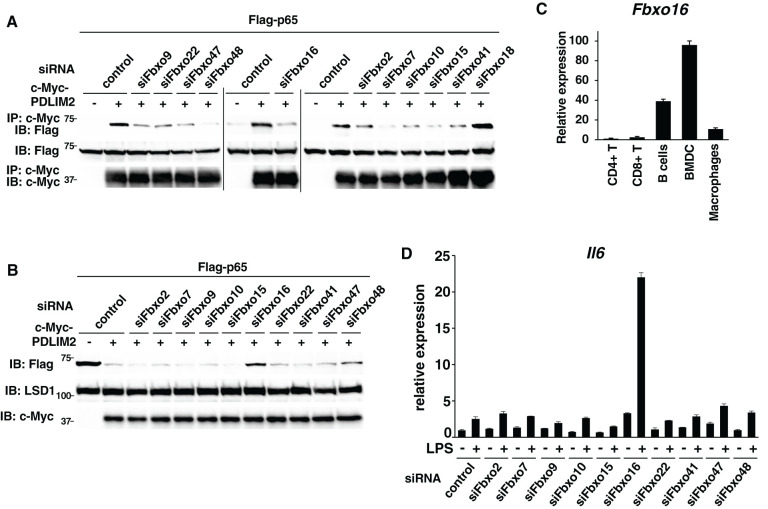
Fbxo16 is a substrate-recognizing component in the PDLIM2-containing ubiquitin E3 ligase complex. **(A)** Effect of deficiency of Fbxo proteins on the interaction of PDLIM2 and p65 in HEK293T cells, first transfected with control siRNA or siRNA-specific to Fbxo9, 22, 47, 48, 16, 2, 7, 10, 15, 41 or 18 and then transfected with Flag-p65 without or with c-Myc-PDLIM2. Whole cell lysates were immunoprecipitated with anti-c-Myc and immunoblotted with anti-Flag. **(B)** HEK293T cells first transfected without or with siRNA-specific to Fbxo2, 7, 9, 10, 15, 16, 22, 41, 47 or 48, then transfected with plasmids encoding Flag-p65 without or with c-Myc-PDLIM2. The soluble nuclear extracts were analyzed by anti-Flag antibody. **(C)** Real-time PCR analysis of Fbxo16 expression in immune cells, including CD4^+^T, CD8^+^T, CD19^+^B cells, BMDC and macrophages. **(D)** Effect of deficiency of Fbxo proteins on IL-6 expression in GM-CSF-BMCs transfected with control siRNA or siRNA against Fbxo2, 7, 9, 10, 15, 16, 22, 41, 47 or 48, left untreated or stimulated with LPS (0.1 ng/ml) for 5 h, and analyzed by real-time PCR. Data are representative of three **(A)**, five **(B)**, three (**C**; means ± SD) or four (**D**; means ± SD) independent experiments.

For the first screening, we used a co-immunoprecipitation assay with PDLIM2 and p65. We transfected HEK293T cells with Flag-p65 and c-Myc-PDLIM2 in the absence or presence of siRNA against 39 Fbxo proteins (**Supplementary Table 1**), immunoprecipitated them with an anti-c-Myc beads, and immunoblotted with anti-Flag to determine if siRNA targeting any of these Fbxo proteins could disrupt the binding between p65 and PDLIM2. Among the Fbxo proteins, knockdown of 10 FBXO family members, including Fbxo2, 7, 9, 10, 15, 16, 22, 41, 47 and 48, impaired the association of PDLIM2 with p65 (**Figure 2A**). Note that Fbxo18 knockdown did not inhibit this interaction. For the second more stringent screening, we tested if knockdown of any of these ten candidate FBXO proteins interferes with PDLIM2-dependent p65 degradation. We transfected HEK293T cells with Flag-p65 and c-Myc-PDLIM2 in the absence or presence of siRNA specific to these ten FBXO proteins and measured p65 protein levels in the soluble nuclear fraction. The reduction of mRNA encoding each FBXO protein in cells transfected with each FBXO protein-specific siRNA was confirmed by real-time PCR analysis (**Supplementary Figure 4**) using the primers shown in **Supplementary Table 2**. PDLIM2 overexpression markedly decreased p65 protein in this fraction, whereas only Fbxo16 knockdown clearly reverted the PDLIM2-dependent decrease in p65 protein levels (**Figure 2B**), suggesting that Fbxo16 is most likely to be the substrate-recognizing subunit in the PDLIM2-containing CRL1 complex.

We then examined the expression of Fbxo16 in immune cells and found that it is expressed at highest levels in dendritic cells (BMDC) but at very low levels in CD4^+^ and CD8^+^ T cells (**Figure 2C**). We have previously shown that PDLIM2 negatively regulates TLR-mediated p65-activation in dendritic cells, so that PDLIM2-deficient dendritic cells have enhanced TLR-mediated inflammatory cytokine production (11). For the final screening, we have therefore examined if knockdown of any of the ten candidate FBXO proteins in dendritic cells augmented LPS-induced IL-6 expression. We transfected GM-CSF-differentiated bone marrow cells (GM-CSF-BMCs) with siRNA specific to the candidate FBXO proteins (**Supplementary Table 3**), stimulated cells with LPS and examined IL-6 expression by real-time PCR analysis. The efficiency of knockdown by each FBXO protein was determined by real-time PCR analysis (**Supplementary Figure 5**) using specific primers shown in **Supplementary Table 4**. We could detect a striking augmentation of LPS-induced IL-6 expression only in Fbxo16 knockdown dendritic cells compared to control cells (**Figure 2D**). Taking these data together, we speculated that Fbxo16 is the best candidate for the substrate-recognizing receptor for p65 in the PDLIM2-containing CRL1 complex.

### 3.3 Fbxo16 forms a CRL1 complex, binds to p65 and negatively regulates NF-κB signaling

**Figure 3:**
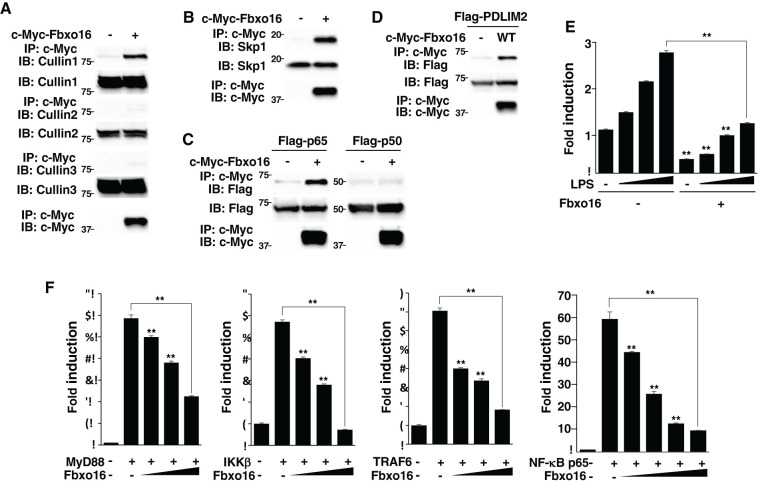
Fbxo16 forms a CRL1 complex, binds to p65 and negatively regulates NF-κB signaling. **(A)** Interaction of Fbxo16 and Cullins in whole cell lysates of HEK293T cells, transfected with c-Myc-Fbxo16, then immunoprecipitated with anti-Flag, and immunoblotted with anti-Cullin 1, Cullin 2 or Cullin 3. **(B)** Interaction of Fbxo16 and Skp1 in whole cell lysates of HEK293T cells, transfected with c-Myc-Fbxo16, then immunoprecipitated with anti-c-Myc, and immunoblotted with anti-Skp1. **(C)** Interaction of Fbxo16 and p65 in whole cell lysates of HEK293T cells, transfected with a Flag-p65 or p50 along without or with c-Myc-Fbxo16, then immunoprecipitated with anti-c-Myc, and immunoblotted with anti-p65 or p50. **(D)** Interaction of Fbxo16 and PDLIM2 in whole cell lysates of HEK293T cells, transfected with a Flag-PDLIM2 along without or with c-Myc-Fbxo16, then immunoprecipitated with anti-c-Myc, and immunoblotted with anti-Flag. **(E)** Luciferase assay in MEFs transfected with ELAM−1 luciferase reporter and pRL-Null renilla constructs along with increasing amounts (wedge) of Fbxo16, then left untreated or stimulated with LPS for 5 hr. **(F)** Luciferase assay in HEK293T cells transfected with ELAM−1 luciferase reporter and pRL-Null renilla constructs without or with MyD88, IKKβ, TRAF6 or p65 along with increasing amounts (wedge) of Fbxo16. Data are representative of three **(A, B)**, four **(C, D)** or three (**E, F**; means ± SD, **P<0.01) independent experiments.

To demonstrate that Fbxo16 is a substrate p65-recognizing subunit in the PDLIM2-containing CRL1 complex, we first examined the association of Fbxo16 with the other components in the complex. HEK293T cells were transfected with c-Myc-Fbxo16, immunoprecipitated with anti-c-Myc beads, and immunoblotted with antibodies against Cullin 1, Cullin 2, Cullin 3 and Skp1. We demonstrated that Fbxo16 binds to Cullin1 and Skp1, but not Cullin2 or Cullin3 (**Figures 3A, B**), which is compatible with the general feature of F-box proteins that forms a CRL1 complex through Skp1. We then tested if Fbxo16 interacted with p65 and found that Fbxo16 could bind to p65, but not to p50, component of NF-κB (**Figure 3C**). Moreover, we have also shown by a co-immunoprecipitation assay that Fbxo16 binds to PDLIM2 (**Figure 3D**). These data demonstrated that Fbxo16 can physically interact with all the components in PDLIM2-containing CRL1 complex.

Since PDLIM2 inhibits TLR-mediated NF-κB activation (11), we next tested the effect of Fbxo16 on TLR-mediated, NF-κB-induced transactivation in a luciferase assay. MEFs were transfected with the ELAM-1 luciferase reporter that can be driven by NF-κB, and then stimulated with LPS for 5 hr. LPS stimulation increased luciferase activity, while coexpression of Fbxo16 markedly inhibited transactivation of the LPS-induced luciferase reporter (**Figure 3E**). In contrast to the nuclear localization of PDLIM2, Fbxo16 is localized in both the cytoplasm and the nucleus (https://www.proteinatlas.org/), suggesting that Fbxo16 might target upstream cytoplasmic signaling molecules in addition to p65. To rule out this possibility, we cotransfected HEK293T cells with p65 or upstream cytoplasmic signaling molecules MyD88, TRAF6, or IKKβ to activate the ELAM-1 luciferase reporter, without or with Fbxo16, and examined if Fbxo16 could affect gene activation driven by these molecules. Fbxo16 inhibited MyD88-, TRAF6-, IKKβ-, and p65-mediated NF-κB activation to almost the same extent (**Figure 3F**). These data suggest that Fbxo16 acts on p65, the most downstream molecule, to inhibit NF-κB signaling.

### 3.4 Fbxo16 mediates the polyubiquitination and degradation of p65 through its F-box domain

**Figure 4:**
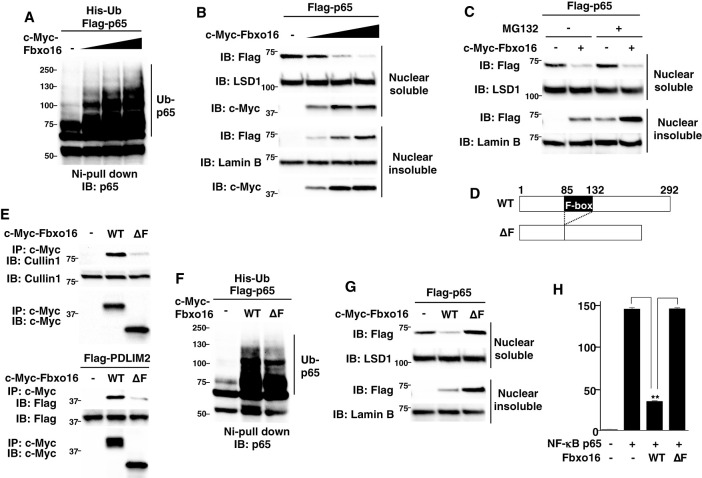
Fbxo16 mediates the polyubiquitination and degradation of p65 through its F-box domain. **(A)** Ubiquitination assay of p65 in HEK293T cells transfected with His-ubiquitin (His-Ub), p65 and increasing amounts (wedge) of Fbxo16. The purified ubiquitinated proteins were analyzed by immunoblot with anti-p65. **(B)** Effect of Fbxo16 on soluble and insoluble nuclear p65 in HEK293T cells transfected with Flag-p65 and increasing amounts (wedge) of c-Myc-Fbxo16, analyzed by immunoblot with ant-Flag antibody. **(C)** Effect of proteasome inhibitor on PDLIM2-mediated p65 degradation in soluble and insoluble nuclear extracts of HEK293T cells, transfected with Flag-p65 without or with c-Myc-Fbxo16, then left untreated or treated for 4 h with MG132 (10μM) and analyzed by immunoblot with anti-Flag antibody. **(D)** The structure of the wild-type Fbxo16 protein and Fbxo16 mutant lacking the F-box domain (ΔF). **(E)** Effect of the deletion of F-box domain on the interaction of Fbxo16 and Cullin 1 (top) or PDLIM2 (bottom) in HEK293T cells, transfected without or with c-Myc-wild-type or ΔF Fbxo16, immunoprecipitated with anti-c-Myc and immunoblotted with anti-Cullin 1 antibody (top), or transfected with Flag-PDLIM2 along with or without c-Myc-wild-type or ΔF Fbxo16, immunoprecipitated with anti-c-Myc and immunoblotted with anti-Flag antibody (bottom). **(F)** Ubiquitination assay of p65 in HEK293T cells cotransfected with His-ubiquitin, p65 along without or with wild-type or ΔF Fbxo16. The purified ubiquitinated proteins were analyzed by immunoblot with anti-p65. **(G)** Effect of the deletion of F-box domain on Fbxo16-mediated p65 degradation in soluble and insoluble nuclear extracts of HEK293T cells transfected with Flag-p65, along without or with wild-type or ΔF Fbxo16 and analyzed by immunoblot with anti-Flag antibody. **(H)** Luciferase assay in HEK293T cells transfected with ELAM−1 luciferase reporter, pRL-Null renilla constructs and Flag-p65 without or with wild-type or ΔF Fbxo16. Data are representative of three **(A–C**, **E–G)** or three (**H**; means ± SD, **P<0.01) independent experiments.

Since PDLIM2 promoted polyubiquitination of p65 and following its degradation by proteasome (11), we investigated if Fbxo16 also polyubiquitinate p65 and found that Fbxo16 could promote polyubiquitination of p65 in a dose-dependent manner (**Figure 4A**). To assess the activity of Fbxo16 to degrade p65, we next transfected HEK293T cells with Flag-p65 together without or with c-Myc-Fbxo16 and examined the p65 protein in soluble and insoluble nuclear fractions. The p65 protein expression was decreased in the soluble nuclear fraction but increased in the insoluble nuclear fraction by coexpression of Fbxo16 (**Figure 4B**). MG132, a proteasome inhibitor, treatment led to the increase of p65 in the insoluble, but not soluble, nuclear fraction (**Figure 4C**), suggesting that Fbxo16, possibly together with PDLIM2, promotes the transport of p65 to the insoluble nuclear fraction and following proteasomal degradation of p65 in this compartment. To clarify the role of the F-box domain, which is responsible for the binding to Skp1 in the CRL1 complex, we generated a Fbxo16 mutant lacking the F-box domain; ΔF (**Figure 4D**). This mutant was impaired to bind to either Cullin1 or PDLIM2 and to polyubiquitinate p65 (**Figures 4E, F**), indicating that CRL1 complex formation was almost completely disrupted by deletion of the F-box domain. We then transfected HEK293T cells with Flag-p65 together without or with wild-type or ΔF Fbxo16 and examined the p65 protein level in soluble and insoluble nuclear fractions. As with the experiments in **Figure 4B**, wild-type Fbxo16 reduced p65 in the soluble nuclear fraction, whereas Fbxo16-ΔF could not decrease p65 in this fraction, possibly because the transport of p65 from soluble to insoluble fractions was almost completely impaired by this mutation (**Figure 4G**). Moreover, Fbxo16 ΔF transfectants resulted in much more accumulation of insoluble p65 than wild-type transfectants. This can be due to reduced p65 degradation in the insoluble fraction as a consequent of impaired p65 polyubiquitination in Fbxo16-ΔF transfectants. Consistently, the activity of Fbxo16-ΔF to inhibit NF-κB activation in the luciferase assay was completely impaired (**Figure 4H**). These data indicated that Fbxo16, and possibly CRL complex formation itself, is essential for both intranuclear transport of p65 to the insoluble fraction and following p65 degradation in this fraction.

**Figure 5:**
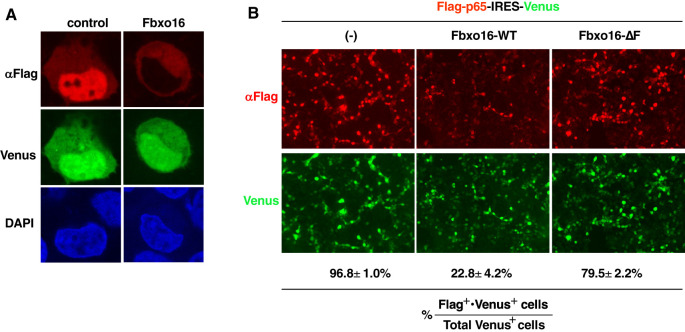
Confocal microscopy of F-box-dependent p65 degradation by Fbxo16 **(A)** Immunofluorescence staining of HEK293T cells transfected with the Flag-p65-IRES-Venus construct without or with c-Myc-Fbxo16 plasmid. Anti-Flag staining (top); Venus expression (middle). The nucleus was stained with DAPI (bottom) **(B)** Immunofluorescence staining of HEK293T cells transfected with the Flag-p65-IRES-Venus construct without or with c-Myc-wild-type or ΔF Fbxo16 plasmid. Anti-Flag staining (top); Venus expression (bottom). The percentage of Flag^+^•Venus ^+^ cells per total Venus^+^ cells was shown. Data are representative of three **(A, B)** independent experiments.

To visualize the effect of Fbxo16 on p65 degradation in the nucleus, we next used a plasmid that bicistronically expresses both Flag-tagged p65 and Venus proteins (Flag-p65-IRES-Venus) and transfected it into HEK293T cells without or with the Fbxo16 expression plasmid. Cells were then examined by indirect immunofluorescence using confocal microscopy. The percentage of Flag^+^•Venus ^+^ or Flag^-^•Venus ^+^ cells per total Venus^+^ cells was calculated to assess the effects of Fbxo16 on nuclear p65. Overexpressed p65 exhibited homogeneous nuclear distribution (**Figure 5A**, left column). On the other hand, Fbxo16 coexpression led to the loss of nuclear p65 staining (**Figure 5A**, right column). In control cells, 96.8 ± 1.0% of Venus-expressing cells also expressed Flag-p65, whereas only 22.8 ± 4.2% of Venus-expressing cells expressed Flag-p65 in Fbxo16 transfectant. Notably, cells with coexpression of Fbxo16-ΔF restored the Flag-p65 expression up to 79.5 ± 2.2% due to impaired ubiquitination-dependent degradation of p65 (**Figure 5B**).

### 3.5 NF-κB-mediated inflammatory responses were enhanced in Fbxo16 deficient dendritic cells

**Figure 6:**
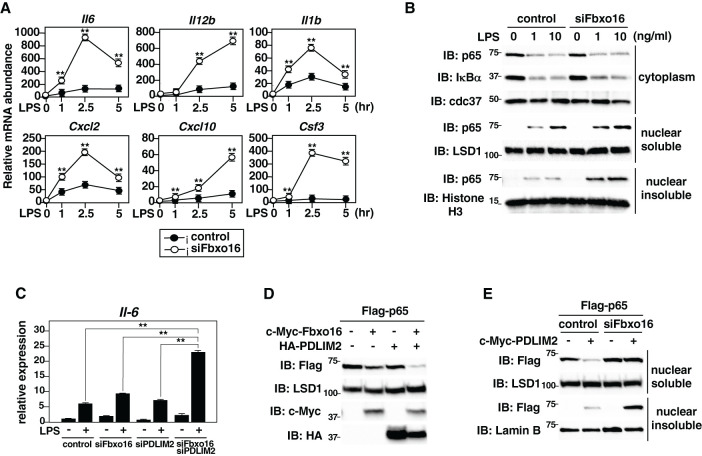
NF-κB-mediated inflammatory responses were enhanced in Fbxo16 deficient dendritic cells. **(A)** Expression of proinflammatory cytokine genes in GM-CSF-BMCs transfected with control siRNA or Fbxo16 against siRNA, stimulated with LPS (0.1 ng/ml) for 1, 2.5 and 5 hr, and analyzed by real-time PCR. **(B)** Effect of Fbxo16 deficiency on cytoplasmic and soluble and insoluble nuclear p65 in GM-CSF-BMCs transfected with control siRNA or Fbxo16 against siRNA, then stimulated with 0, 1, or 10 ng/ml of LPS for 1 h and analyzed with indicated antibodies. **(C)** Effect of Fbxo16 and/or PDLIM2 deficiency on IL-6 expression in GM-CSF-BMCs transfected with control siRNA or siRNA against Fbxo16 and/or PDLIM2, then stimulated with 0 (-) or 0.1 (+) ng/ml of LPS for 2 h and analyzed by real-time PCR. **(D)** Synergistic effect of Fbxo16 and PDLIM2 expression on soluble nuclear p65 in HEK293T cells transfected with Flag-p65, together with c-Myc-tagged Fbxo16 and/or HA-tagged PDLIM2 in the indicated combination and analyzed by immunoblot with anti-Flag antibody. **(E)** Effect of Fbxo16 deficiency on soluble and insoluble nuclear p65 in HEK293T cells first transfected with control siRNA or siRNA against Fbxo16, transfected with Flag-p65 without or with c-Myc-PDLIM2 and analyzed by immunoblot with anti-Flag antibody. Data are representative of four (**A**; means ± SD, **P<0.01), three **(B)**, three (**C**; means ± SD, **P<0.01) or three **(D, E)** independent experiments.

We next investigate the role of Fbxo16 in the regulation of TLR-mediated NF-κB activation. We knocked down Fbxo16 in GM-CSF-differentiated bone marrow cells (GM-CSF-BMCs) by siRNA, stimulated them with LPS, and analyzed the effect of Fbxo16 deficiency on LPS-induced proinflammatory cytokine gene expression. The expression of IL-6, IL12, IL-1β, CXCL-2, CXCL-10, and G-CSF (*Csf3*) was significantly upregulated in LPS-stimulated Fbxo16 knockdown cells compared to LPS-stimulated control cells at all time points we tested (**Figure 6A**). We then examined the effect of Fbxo16 knockdown on LPS-induced p65 activation by immunoblot. The amounts of p65 protein in the nuclear soluble and nuclear insoluble, but not cytoplasmic, fractions were increased in LPS-stimulated Fbxo16 knockdown cells compared to LPS-stimulated control cells (**Figure 6B**). The increased nuclear p65 level by Fbxo16 knockdown was quantified using densitometric analysis (**Supplementary Figure 6**). It is of note that the LPS-induced degradation of IκBα, leading to the nuclear translocation of p65, was normal in Fbxo16 knock down cells, indicating that the increased nuclear p65 by Fbxo16 knockdown was due to impaired p65 degradation in the nucleus. These data suggest that Fbxo16 promotes the degradation of nuclear, but not cytoplasmic, p65 protein and negatively regulates NF-κB-mediated inflammatory responses. We next examined the synergistic effects of Fbxo16 and PDLIM2 on LPS-induced IL-6 expression. We knocked down Fbxo16 and/or PDLIM2 in GM-CSF-BMCs at a suboptimal condition, in which lower amount of siFbxo16 and/or siPDLIM2 was transfected compared to **Figure 6A**. and examined the expression of IL-6 by real-time PCR analysis. Double knockdown of Fbxo16 and PDLIM2 resulted in augmented production of IL-6 compared to control cells or cells knocked down either Fbxo16 or PDLIM2 (**Figure 6C**). We also examined the synergistic effect of Fbxo16 and PDLIM2 on p65 degradation in the nucleus. We transfected HEK293T cells with Flag- p65 along with c-Myc-Fbxo16 and/or HA-PDLIM2 in a suboptimal condition, in which lower amounts of Fbxo16 and PDLIM2 were transfected compared to **Figures 2B**, **4C**. Overexpression of either Fbxo16 or PDLIM2 barely reduced p65 levels in the soluble nuclear fraction, whereas the coexpression of Fbxo16 plus PDLIM2 markedly decreased p65 protein. (**Figure 6D**). To further confirm the role of Fbxo16 in PDLIM2-mediated degradation p65 protein, we transfected HEK293T cells with Flag-p65 together without or with c-Myc-Fbxo16 and examined the p65 protein level in soluble and insoluble nuclear fractions in the absence or presence of siRNA against Fbxo16. Fbxo16 knockdown completely restored the PDLIM2-mediated decrease of soluble nuclear p65 to the original level and led to the accumulation of insoluble nuclear p65 protein (**Figure 6E**). Taking these data together, we concluded that Fbxo16 and PDLIM2 form a complex and synergistically promotes the intranuclear transport of p65 to the insoluble fraction and its degradation in this compartment, thereby suppressing inflammatory responses.

### 3.6 Fbxo16 does not promote polyubiquitination and degradation of STAT3/4

**Figure 7:**
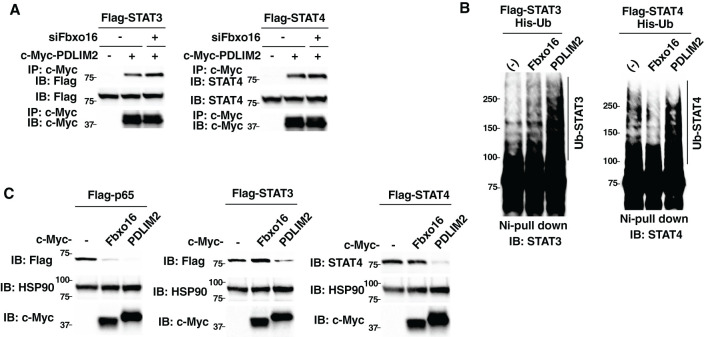
Fbxo16 does not promote polyubiquitination and degradation of STAT3/4. **(A)** Effect of Fbxo16 deficiency on the interaction of PDLIM2 and STAT3 (left) or STAT4 (right) in HEK293T cells, transfected Flag-STAT3 (left) or Flag-STAT4 (right) together without or with c-Myc-PDLIM2, then immunoprecipitated with anti-c-Myc and immunoblotted with anti-Flag (left) or anti-STAT4 (right). **(B)** Ubiquitination assay of STAT3/4 in 293T cells transfected with His-Ub, Flag-STAT3 (left) or Flag-STAT4 (right) without or with c-Myc Fbxo16 or PDLIM2. The purified ubiquitinated proteins by Ni-NTA beads were analyzed with anti-STAT3 (left) or anti-STAT4 (right) antibodies, respectively. **(C)** Effect of Fbxo16 or PDLIM2 expression on the degradation of p65 (left), STAT3 (middle) or STAT4 (right) in whole cell lysates of HEK293T cells transfected with Flag-p65 (left), STAT3 (middle) or STAT4 (right) along without or with c-Myc-Fbxo16 or PDLIM2 and analyzed with anti-Flag (left and middle) or anti-STAT4 (right) antibodies. Data are representative of three **(A-C)** independent experiments.

We next examined the substrate specificity of Fbxo16. Since PDLIM2 binds to and promote polyubiquitination and degradation of STAT3/4 as well as p65 (9, 12), we first tested if Fbxo16 knockdown affected the ability of PDLIM2 to bind to STAT3/4. We transfected HEK293T cells with Flag-tagged STAT3 or STAT4 and c-Myc-tagged PDLIM2 in the absence or presence of siRNA against Fbxo16, immunoprecipitated with an anti-c-Myc beads, and immunoblotted with anti-Flag or anti-STAT4 antibody. In contrast to the impaired interaction between p65 and PDLIM2 in Fbxo16 knockdown cells (**Figure 2A**), Fbxo16 knockdown did not interfere with the binding of PDLIM2 to either STAT3 or STAT4 (**Figure 7A**, **Supplementary Figure 7**). We next examined if PDLIM2 promoted polyubiquitination and degradation of STAT3/4. As shown in **Figure 7B**, Fbxo16 did not lead to the ubiquitination of either STAT3 or STAT4. Moreover, overexpression of PDLIM2, but not Fbxo16, promoted degradation of STAT3 and STAT4, although p65 could be degraded by either Fbxo16 or PDLIM2 (**Figure 7C**).

### 3.7 Enhanced p65-mediated inflammatory responses, but normal Th1 and Th17 differentiation in Fbxo16-deficient mice

**Figure 8:**
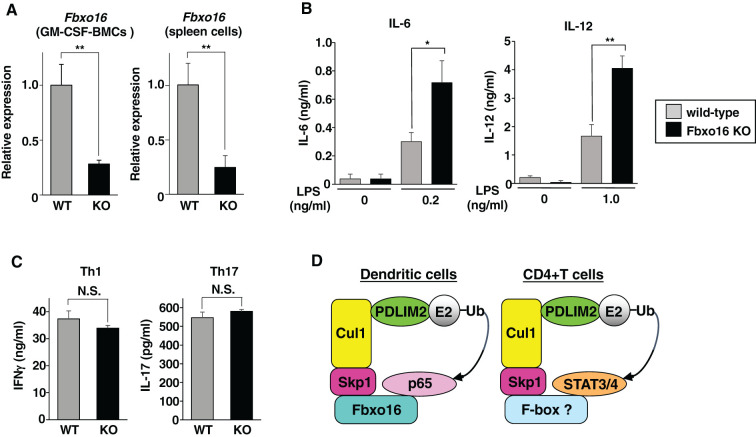
Enhanced p65-mediated inflammatory responses, but normal Th1 and Th17 differentiation in Fbxo16-deficient mice. **(A)** Fbxo16 expression in GM-CSF-BMCs and total spleen cells from *Fbxo16^++/^* (WT; gray bar) and *Fbxo16^-/-^* (KO; black bar) mice, analyzed by real-time PCR. **(B)** IL-6 (left) and IL-12 (right) production in culture supernatants of GM-CSF-BMCs from *Fbxo16^++/^* and *Fbxo16^-/-^* mice, stimulated for 24 h with the indicated concentration of LPS and analyzed by ELISA. **(C)** CD4^+^T cells, isolated from the spleen of *Fbxo16^++/^* and *Fbxo16^-/-^* mice, were differentiated under Th1 and Th17-skewing conditions for 4 days and then restimulated with plate-bound anti-CD3 antibody for 24 (h) IFNγ (left) and IL17 (right) production in culture supernatants were analyzed by ELISA. **(D)** The mechanisms by which Fbxo16 in the PDLIM2-containing CRL1 complex acts as substrate-recognizing receptor for p65 in dendritic cells, but not for STAT3/4 in CD4^+^T cells. Data are representative of three (**A**; means ± SD, **P<0.01), four (**B**; means ± SD, *P<0.05, **P<0.01) or three (**C**; N.S., not significant) independent experiments.

Finally, to investigate the *in vivo* roles of Fbxo16, we generated Fbxo16-deficient mice by the CRISPR-Cas9 system (**Supplementary Figure 1**). Since the antibodies that can sufficiently detect endogenous Fbxo16 protein are not available, we analyzed expression of Fbxo16 mRNA in *Fbxo16^-/-^* GM-CSF-BMCs and spleen cells by real-time PCR. Compared to *Fbxo16^+/+^* cells, Fbxo16 mRNA expression was reduced in both *Fbxo16^-/-^* GM-CSF-BMCs and spleen cells (**Figure 8A**), which might be ascribed to nonsense-mediated mRNA decay due to the newly generated premature termination codon just after the deleted region (25). Moreover, even if truncated Fbxo16 protein could be expressed at a very low level, it would not be functional because almost the entire region of the F-box domain, which is essential for Fbxo16 activity (**Figures 4E-H**), is missing due to the CRISPR-Cas9-mediated deletion. We then examined the LPS-induced production of proinflammatory cytokine by *Fbxo16^-/-^* GM-CSF-BMCs. The LPS-stimulated GM-CSF-BMCs from *Fbxo16^-/-^* mice produced two- to threefold more IL-6 and IL-12 than the LPS-stimulated *Fbxo16^+/+^* cells (**Figure 8B**). We next examined the differentiation of CD4^+^T cells into Th1 and Th17 cells driven by the IL-12-mediated STAT4 or IL-6-mediated STAT3 activation of transcription factors, respectively, and found that both Th1 and Th17 cell differentiation were unaffected in the *Fbxo16^-/-^* CD4^+^T cells (**Figure 8C**), suggesting that Fbxo16 specifically regulate p65-dependent NF-κB-signaling in GM-CSF-BMCs, but not STAT3/4-mediated T-helper cell differentiation in CD4^+^T cells. Taking these data together, we propose the model that Fbxo16 functions as a substrate-recognizing receptor for p65 in the PDLIM2-containing CRL1 complex, thereby mediating the degradation of p65 in dendritic cells but does not act as substrate receptor for STAT3/4 in CD4^+^T cells (**Figure 8D**).

## 4 Discussion

NF-κB activation is essential for in dendritic cells to induce inflammatory responses and initiate T cell-mediated acquired immune responses. On the other hand, excessive and persistent activation of NF-κB may lead to autoimmune and allergic diseases. Notably, activated NF-κB is detected at the regions of inflammation in these diseases (3). The NF-κB activation should therefore be strictly controlled by negative regulatory systems. Recently, the molecular mechanisms negatively regulating NF-κB signaling have been intensively studied (26, 27). Many factors that suppress various signal transduction molecules, from the receptor on the cell surface to the promoter on the DNA, have already been identified. The ubiquitin-dependent degradation of p65 subunit of NF-κB is one of the most efficient mechanism terminating NF-κB signaling. So far, several ubiquitin E3 ligases for p65 have been reported, including SOCS1 (suppressor of cytokine signaling 1) (28), COMMD1 (COMM domain containing 1) (29), PPARγ (peroxisome proliferator-activated receptor-γ) (30), ING4 (inhibitor of growth protein 4) (31), MKRN2 (makorin ring finger protein 2) (32), RNF182 (RING finger protein 182) (33), RBCK1 (RanBP2-type and C3HC4-type zinc finger-containing 1) (34), TRIM7 (tripartite motif-containing 7) (35) and LRSAM1 (leucine-rich and sterile alpha motif containing 1) (36). PPARγ, TRIM7, LRSAM1, RBCK1 and RNF182 have RING finger domain, whereas ING4 contains a PHD domain, a subtype of the RING domain. Notably, SOCS1 and COMMD1 form a CRL2 complex with Cullin 2, Elongin B/C and a RING finger domain-containing protein, Rbx1. In this complex, SOCS1 acts as a substrate-recognizing subunit for p65 and COMMD1 stabilizes the association between SOCS1 and p65 (29).

We have reported for the first time that PDLIM2 is a nuclear ubiquitin E3 ligase mediating polyubiquitination and degradation of the p65 subunit of NF-κB in the nucleus, thereby negatively regulating inflammatory responses in dendritic cells (11). However, the detailed molecular mechanisms by which PDLIM2 recognizes and polyubiquitinates target proteins remained unclear. In this study, we have demonstrated that PDLIM2 forms a CRL1 complex with Cullin 1 and Skp1 and identified Fbxo16, an F-box protein that belongs to the FBXO subfamily, is the substrate-recognizing subunit of this complex. Fbxo16 binds to all the components of this complex, including Cullin 1, Skp1 and PDLIM2, recruits p65 and then promotes polyubiquitination and degradation of p65 just as PDLIM2 does. Moreover, Fbxo16 deficiency in dendritic cells led to increased nuclear p65 and enhanced TLR-induced production of proinflammatory cytokines, including IL-6 and IL-12, a phenotype identical to PDLIM2-deficient dendritic cells (11). Taking these data together, we concluded that Fbxo16 is the substrate receptor for p65 in PDLIM2-containing CRL1 complex. Notably, this phenotype of Fbxo16 deficiency was evident in GM-CSF BMCs but not in splenic CD11c+ cells (data not shown). Dendritic cells consist of distinct subsets. The splenic CD11c+ cells are tissue resident DC, whereas GM-CSF BMCs might be equivalent of monocyte-derived DC (inflammatory DC), which can be elicited by infection or inflammation (37). These data suggest that Fbxo16 might be functional in inflammatory DC but not in resident DC. On the other hand, Fbxo16 could not promote polyubiquitination and degradation of either STAT3 or STAT4. Consistently, Fbxo16 deficiency in CD4^+^T cells did not affect IL-6 or IL12-mediated STAT3 or STAT4-dependent Th1 and Th17 cell differentiation, respectively. These data suggest that Fbxo16 is the specific substrate receptor for p65, but not STAT3 or STAT4, in the complex (**Figure 8D**). We predict that another F-box protein should be responsible for recognizing and recruiting STAT3 and STAT4 in the PDLIM2-containing CRL1 complex, although further study will be needed to clarify this issue.

The LIM domain is thought to be a subtype of a RING finger domain since it is structurally related to a RING finger domain (17). Both the LIM domain and RING finger domains commonly have eight conserved cysteine (Cys) or histidine (His) residues, which are critical for the ubiquitin E3 ligase activity of these domains. We have reported for the first time that the protein containing LIM domain functions as ubiquitin E3 ligase (9). This is clearly evidenced by our result showing that the purified recombinant PDLIM2 protein synthesized in *E. coli* has autoubiquitination activity *in vitro*, which is a hallmark of ubiquitin E3 ligase activity (9). Moreover, PDLIM2 polyubiquitinate p65 in LIM domain-dependent manner (11). Among a larger LIM protein family, PDLIM2 belongs to a PDZ-LIM protein subfamily that consists of seven members (PDLIM1-7) containing one PDZ domain and one or three LIM domains in the molecule (38). Notably, even though all these PDZ/LIM proteins contain a LIM domain, only PDLIM2, 6 and 7 have ubiquitin ligase activity (15, 39). The RING finger and LIM domains are thought to be an E2 enzyme-recruiting subunit, which facilitates ubiquitin transfer onto the substrate. Although LIM domains of all PDZ/LIM proteins commonly have eight conserved residues of cysteine and histidine, the homology of their amino acid sequence in regions other than in these conserved residues in the LIM domains is relatively low. Possibly due to the structural differences caused by the differences in these amino acid sequences in the LIM domain, only the LIM domains of PDLIM2, 6 and 7 can bind to E2 enzyme and possess ubiquitin E3 ligase activity. Since the LIM domain was originally considered to be protein-protein interaction domain (40), LIM proteins may have different functions depending on the molecules with which they can interact through their LIM domain. In fact, PDLIM4 binds to and recruits protein tyrosine phosphatase through its LIM domain and also binds to STAT3, promoting dephosphorylation of a phosphotyrosine on STAT3, which is essential for its activation, thereby suppressing STAT3-mediated signaling (our unpublished data). Taken together, we hypothesize that not all LIM proteins have ubiquitin E3 ligase activity and a subset of LIM proteins, in which the LIM domain might be highly structurally related to a RING finger domain, should function as ubiquitin E3 ligases.

We have previously reported that PDLIM2 promotes the intranuclear transport of p65 through its PDZ domain and the proteasomal degradation of p65 in the insoluble nuclear compartment through its LIM domain (11). Consistently, a PDZ domain-deletion mutant of PDLIM2 impaired intranuclear transport of p65 but normally polyubiquitinated p65, whereas a LIM domain-deletion mutant of PDLIM2 failed to elicit polyubiquitination and degradation of p65 but could mediate intranuclear transport of p65 to insoluble nuclear compartments. In this study, we have demonstrated that either Fbxo16 knockdown or deletion of its F-box domain not only led to the accumulation of p65 in the insoluble nuclear fraction but also completely restored the PDLIM2-mediated decrease of p65 in the soluble nuclear fraction (**Figures 4G**, **6E**). This can be ascribed to both impaired intranuclear transport of p65 and insufficient p65 degradation in the insoluble nuclear fraction. In contrast, in our previous study, the knockdown of either HSP70 or BAG1 led to the increased p65 protein in the insoluble nuclear fraction but could not restore the PDLIM2-mediated decrease of p65 in the soluble nuclear fraction (14), suggesting that only p65 degradation in the insoluble nuclear compartment was impaired by these knockdowns. This is probably because both HSP70 and BAG1 are essential for facilitating the association of the p65-PDLIM2 complex with the proteasome leading to the degradation of p65 but are not required for the intranuclear transport of p65 to the insoluble nuclear compartments. Considering that CRL1 complex formation was disrupted by either Fbxo16 knockdown or deletion of the F-box domain from Fbxo16 (**Figures 2A**, **4E**), the complex formation itself might be critical for the PDLIM2-mediated intranuclear transport.

So far, 69 human and 79 murine F-box proteins have been reported (21, 22). These F-box proteins commonly bind to Cullin 1 or Cullin 7 through their F-box domain and should function as substrate-recognizing subunits in the CRL1 or CRL7 complex (19, 20). F-box proteins exert a variety of functions depending on the substrates that they bind to and recruit to the complex. The substrates for many, but not all, F-box proteins have been identified and most of them were found to be the cell cycle regulators or oncogenes, so that the deficiency of these F-box proteins in human and mice enhanced cell proliferation leading to tumorigenesis (41). In contrast, only several F-box proteins, including Fbxw1, Fbxl2, Fbxo6 and Fbxo38 (42–45), have been reported to be involved in the immune regulation. Regarding Fbxo16, previous reports showed that it mediates polyubiquitination and degradation of β-catenin and heterogeneous nuclear ribonucleoprotein L (hnRNPL), both of which regulate signaling pathways leading to cell proliferation and tumor progression (46–48). Forced expression of Fbxo16 in tumor cell lines inhibited cell growth, whereas knockdown of Fbxo16 in these cells resulted in increased β-catenin and hnRNPL protein levels and enhanced tumor cell proliferation. Notably, the association of higher Fbxo16 expression with better prognosis in cancer patients and the attenuated Fbxo16 expression in higher-grade cancer samples were observed in a human cancer database (47, 48). These data suggest that Fbxo16 acts as a tumor suppressor by inhibiting the activity of β-catenin and hnRNPL. However, the role of Fbxo16 in the immune system remained completely unknown. Here we have demonstrated that Fbxo16 is essential for terminating NF-κB-mediated inflammatory responses by promoting polyubiquitination and degradation of the p65 subunit of NF-κB as the substrate-recognizing receptor for p65 in the PDLIM2-containing CRL1 complex. This is the first report showing that Fbxo16 can regulate signaling pathway in the immune system. Notably, aberrant and persistent activation of NF-κB may promote tumor progression (49), so that the activity of Fbxo16 to inhibit NF-κB activation, which we demonstrated in this study, might be related to the tumor suppressive function of Fbxo16 described above. Fbxo16 could therefore acts as the substrate-recognizing receptor for several proteins involved in cell proliferation, including β-catenin, hnRNPL and NF-κB p65, promotes their degradation and prevents cancer progression (46–48). A recent report showed that Fbxw2 also promotes polyubiquitination and degradation of p65 (50). Although this report did not analyze the complex formation and its role in the immune system, Fbxw2 may be another substrate-recognizing receptor for p65 in the CRL1 complex, thereby regulating NF-κB-mediated inflammatory responses in dendritic cells.

Persistent activation of NF-κB is detected at regions of inflammation in certain human diseases, such as inflammatory bowel diseases and rheumatoid arthritis (3, 4). We found that Fbx16 expression in dendritic cells was suppressed by inflammatory stimuli, such as LPS or CpG DNA (**Supplementary Figure 8**). This attenuated Fbxo16 expression under inflammatory condition may contribute to constitutive NF-κB activation leading to inflammatory diseases. We therefore speculate that Fbxo16-deficiency in humans may cause autoimmune diseases and that the Fbxo16/PDLIM2-mediated pathway to terminate NF-κB activation could be the molecular tool for the development of the new therapy of these diseases.

## Data Availability

The original contributions presented in the study are included in the article/**Supplementary Material**. Further inquiries can be directed to the corresponding author.
