# Supplementary material for: Fbxo16 mediates degradation of NF-κB p65 subunit and inhibits inflammatory response in dendritic cells
Source: Front Immunol. 2025 Jun 3;16:1524110. doi: 10.3389/fimmu.2025.1524110 (PMC12170509; doi:10.3389/fimmu.2025.1524110)
Supplement: Supplementary file 1 [file DataSheet1.pdf]

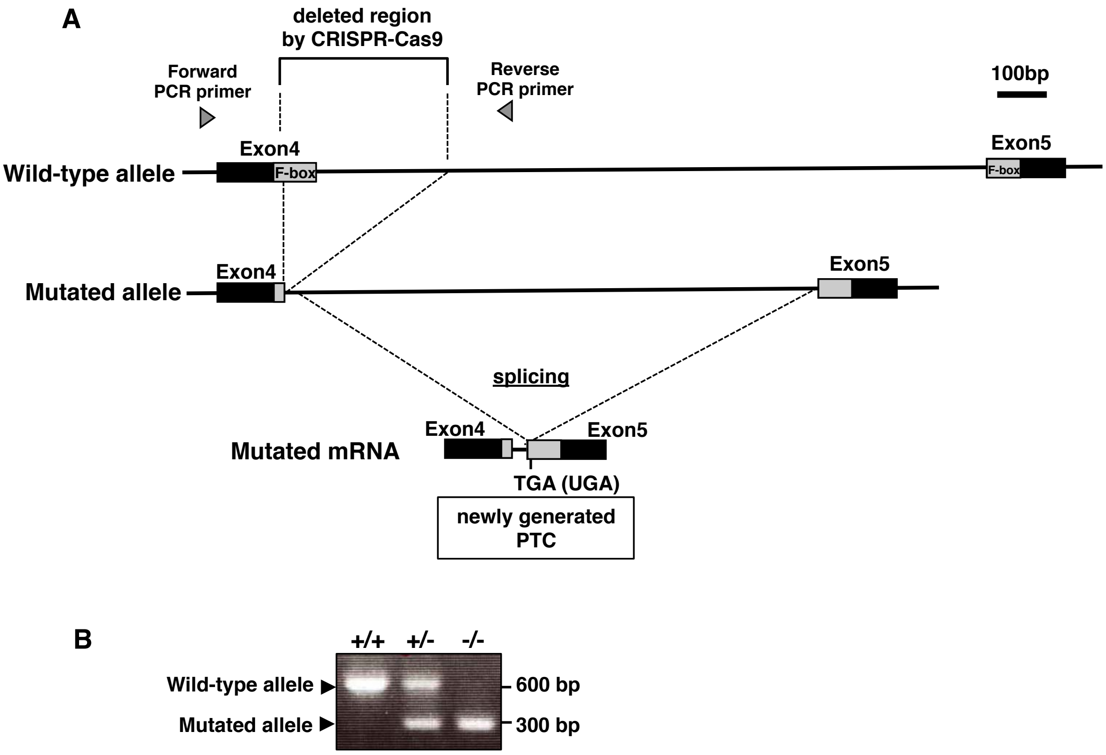

Supplementary Figure 1. Strategy for generating Fbxo16-deficient mice. (**A**) Schematic diagram of the *Fbxo16* wild-type allele and mutated allele in which the latter one-third of exon 4 (68 bp) and a part of intron 4 (287 bp) was deleted by CRISPR-Cas9 system. The exons are shown as black boxes. The coding region of F-box domain is shown as gray box. The arrowheads represent the primers used for genotyping by PCR analysis. The mRNA transcribed from mutated allele was shown at the bottom. The splicing of the mutated exon 4 plus short sequences of intron 4 to exon 5 generates a premature termination codon (PTC) at the beginning of exon 5, resulting in the deletion of almost the entire region of the F-box domain. (**B**) Genotyping of wild-type (+/+), heterozygote (+/-) and homozygote (-/-) mice by PCR using the primer sets shown in **A**. The upper and lower bands were amplified from wild-type and mutated alleles, respectively.

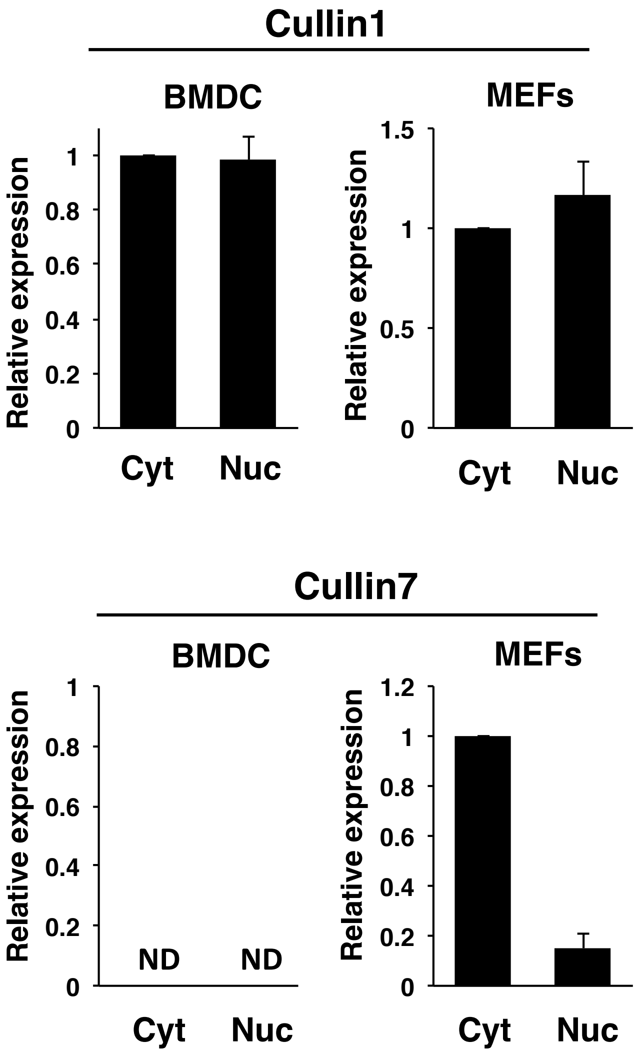

Supplementary Figure 2. Densitometric analysis of nuclear/cytoplasmic expression of Cullin 1 and Cullin 7 in BMDC and MEFs in Figure 1A. Cullin 1 and Cullin 7 protein levels in BMDC and MEFs were normalized against γ1-actin protein level. The ratio of nuclear protein level relative to cytoplasmic protein level was calculated and the mean values ± SD of triplicate experiments were shown. ND: not detected.

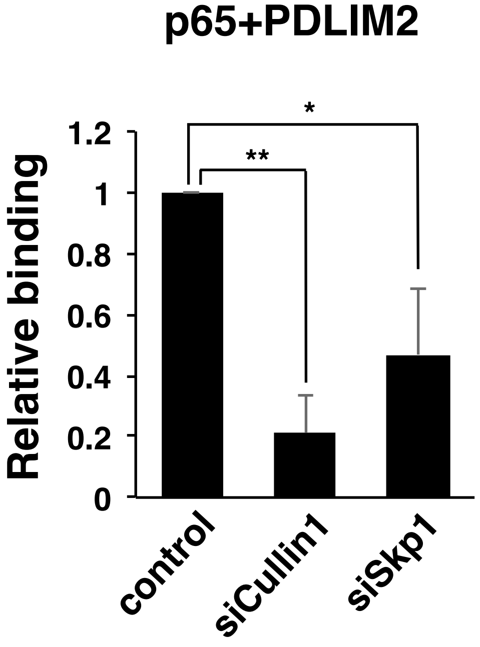

Supplementary Figure 3. Densitometric analysis of the binding of PDLIM2 to p65 in Figure 1F. The coimmunoprecipitated amounts of p65 (Flag expression in IP:c-Myc-IB:Flag) were normalized against the total amounts of p65 (Flag expression in IB:Flag). The percentage decrease in coimmunoprecipitated p65 levels by siCullin1 or siSkp1 treatment relative to control cells was then calculated and mean values ± SD of triplicate experiments are shown. $*P < 0.05$, $**P < 0.01$.

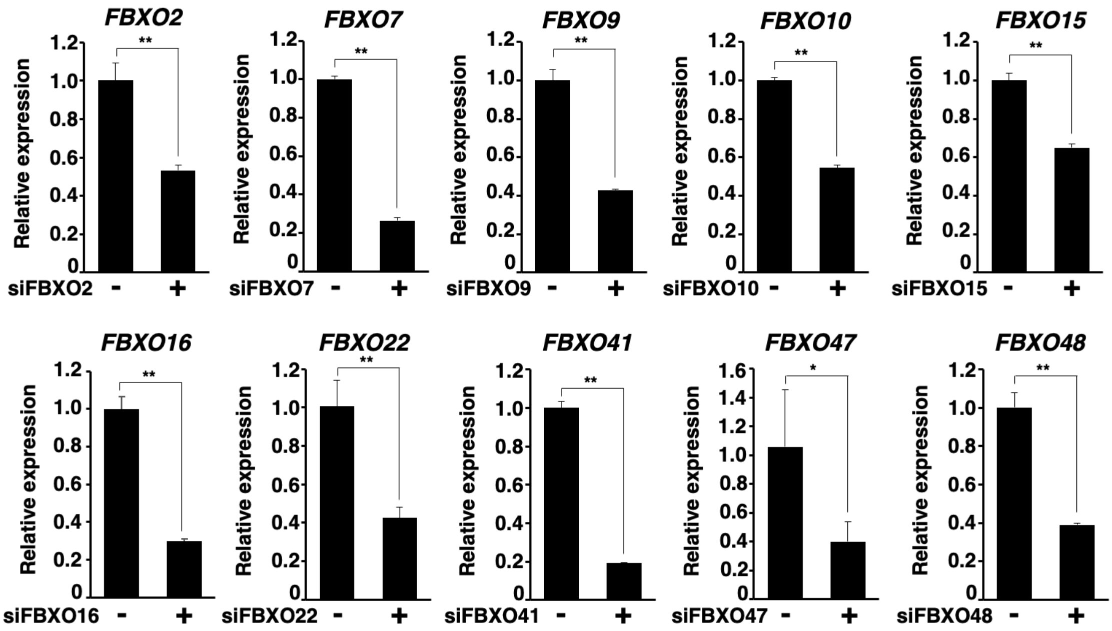

Supplementary Figure 4. **Knockdown efficiency in HEK293T cells.** HEK293T cells transfected with siRNA specific for FBXO2, 7, 9, 10, 15, 16, 22, 41, 47, or 48 were analyzed by real-time PCR analysis to determine the relative expression of mRNAs encoding each indicated FBXO proteins. Data are representative of two independent experiments. Shown are the mean values ± SD. *P<0.05, **P < 0.01.

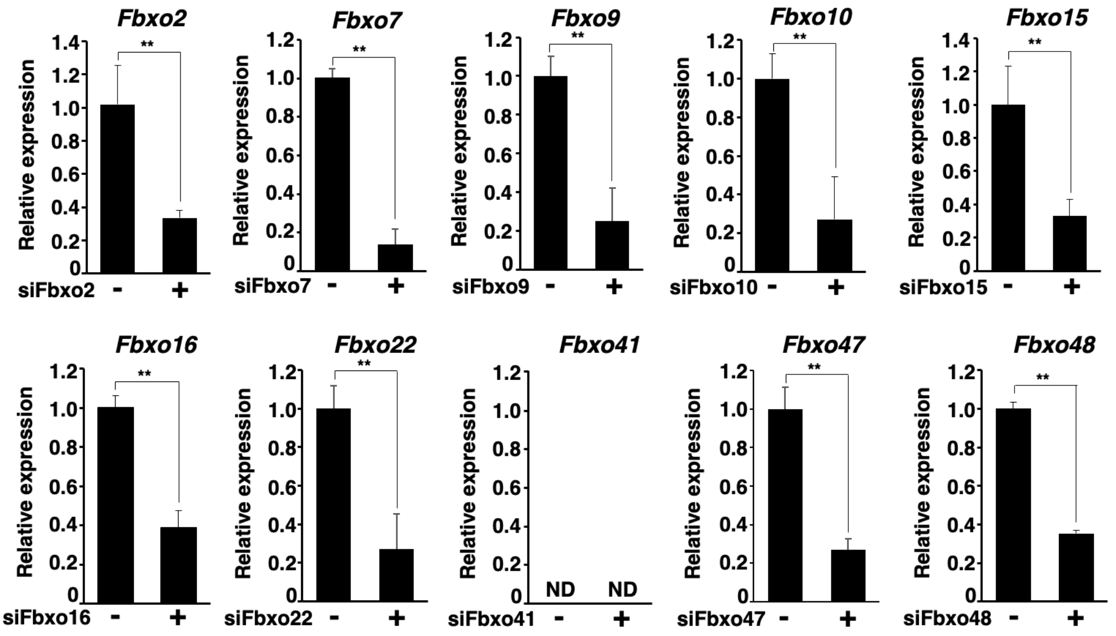

Supplementary Figure 5. **Knockdown efficiency in GM-CSF-BMCs.** GM-CSF-BMCs transfected with siRNA specific for Fbxo2, 7, 9, 10, 15, 16, 22, 41, 47, or 48 were analyzed by real-time PCR analysis to determine relative expression of mRNAs encoding each indicated Fbxo proteins. Note that the expression of Fbxo41 could not be detected (ND) in GM-CSF-BMCs. Data are representative of two independent experiments. Shown are the mean values ± SD. $^{**}P < 0.01$.

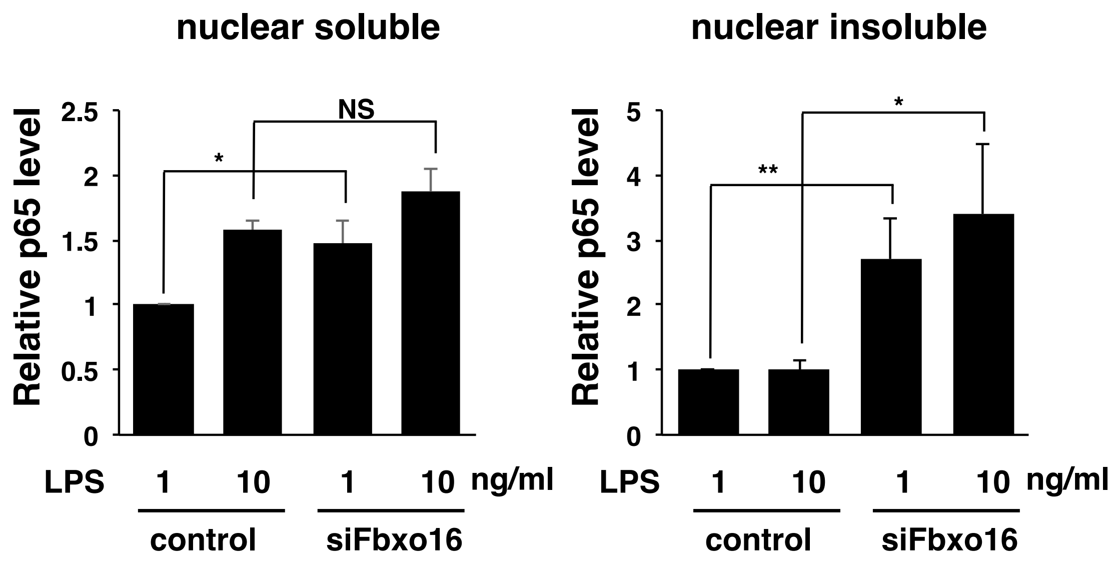

Supplementary Figure 6. Densitometric analysis of the nuclear p65 protein level in control and siFbxo16-treated GM-CSF-BMCs in Figure 6B. The nuclear soluble and insoluble p65 protein levels were normalized against LSD1 (nuclear soluble) or Histone H3 (nuclear insoluble) protein levels. The increase in p65 protein levels relative to control cells stimulated with 1 ng/ml of LPS was calculated and the means values ± SD of triplicate experiments are shown. $^{*}P < 0.05$, $^{**}P < 0.01$, NS: not significant.

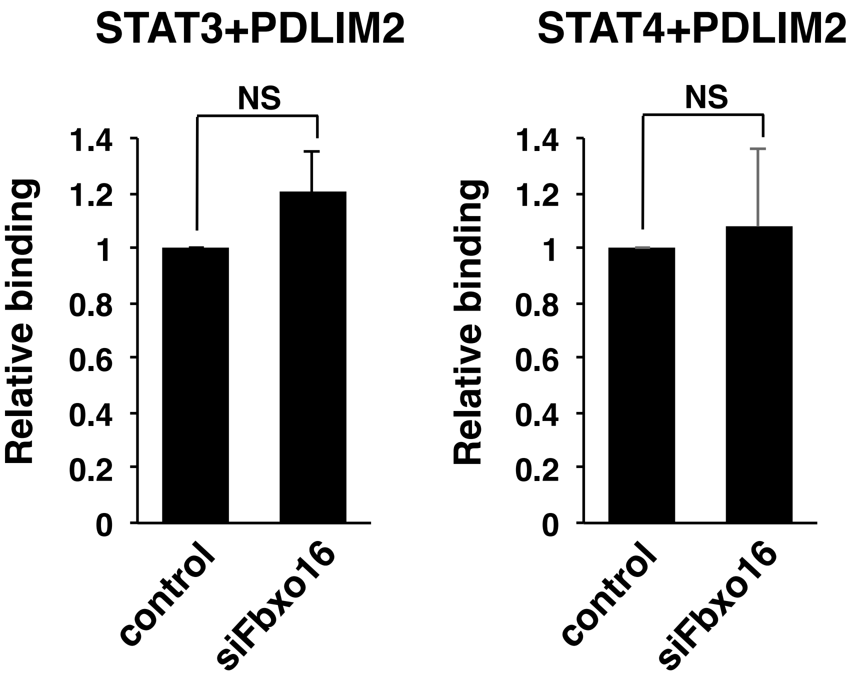

Supplementary Figure 7. Densitometric analysis of the binding of PDLIM2 to STAT3 or STAT4 in Figure 7A. (left panel) The coimmunoprecipitated amounts of STAT3 (Flag expression in IP:c-Myc-IB:Flag) were normalized against the total amounts of STAT3 (Flag expression in IB:Flag). The ratio of coimmunoprecipitated STAT3 level in siFbxo16-treated cells relative to that in control cells was then calculated. (right panel) The coimmunoprecipitated amounts of STAT4 (STAT4 expression in IP:c-Myc-IB:STAT4) were normalized against the total amounts of STAT4 (STAT4 expression in IB:STAT4). The ratio of coimmunoprecipitated STAT4 level in siFbxo16-treatted cells relative that in control cells was then calculated. The mean values ± SD of triplicate experiments are shown. NS: not significant.

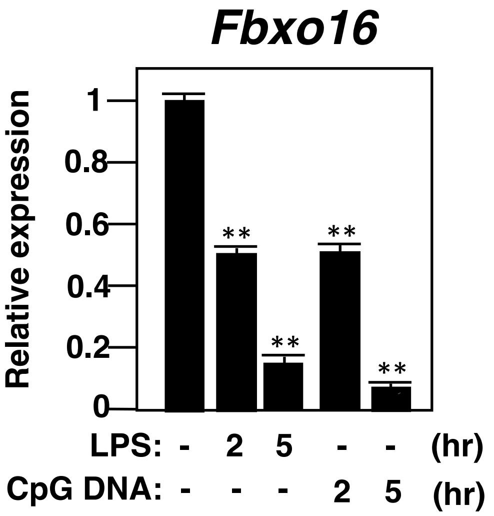

Supplementary Figure 8. **Innate stimuli attenuated Fbxo16 expression in dendritic cells.** Flt3L-differentiated bone marrow-derived dendritic cells (BMDC) were stimulated with LPS (1 ng/ml) or CpG DNA (250 nM) for 2 or 5 hr and analyzed by real-time PCR analysis to determine the relative expression of the mRNA encoding Fbxo16. Data are representative of three independent experiments. Shown are the mean values ± SD. **P < 0.01, compared to unstimulated controls.

Supplementary Table 1. Assay ID of Stealth siRNA for human FBXO proteins

Gene name	siRNA ID	Gene name	siRNA ID	Gene name	siRNA ID
FBXO1	HSS101462	FBXO17	HSS132982	FBXO34	HSS123767
FBXO2	HSS119933	FBXO18	HSS131401	FBXO36	HSS134305
FBXO3	HSS119998	FBXO20	HSS106108	FBXO38	HSS129883
FBXO4	HSS119995	FBXO21	HSS177093	FBXO39	HSS136319
FBXO5	HSS119992	FBXO22	HSS119979	FBXO40	HSS122569
FBXO6	HSS119989	FBXO24	HSS119974	FBXO41	HSS152572
FBXO7	HSS119287	FBXO25	HSS119971	FBXO42	HSS147650
FBXO8	HSS119987	FBXO27	HSS133933	FBXO43	HSS155088
FBXO9	HSS119983	FBXO28	HSS118392	FBXO44	HSS190240
FBXO10	HSS146773	FBXO30	HSS130615	FBXO45	HSS153415
FBXO11	HSS129466	FBXO31	HSS149275	FBXO46	HSS146380
FBXO15	HSS176502	FBXO32	HSS174381	FBXO47	HSS166740
FBXO16	HSS136012	FBXO33	HSS137812	FBXO48	HSS166843

Supplementary Table 2. Primers for human FBXO proteins

Gene name	Primer pairs
FBXO2	Forward: 5'-GTGTCGCAAAGCACAGGTC-3'
	Reverse: 5'-CGGACAGTAGCTTAACGGTGAG-3'
FBXO7	Forward: 5'-CGACAGTATGTTAGGGCCTAGT-3'
	Reverse: 5'-GAGCATGGGTTCTGAGGGATA-3'
FBXO9	Forward: 5'-ACATCGGCAGATACCAAAGGA-3'
	Reverse: 5'- AGCCCTACGATAAAACTTGATGG-3'
FBXO10	Forward: 5'-AGCATTACCTTGCATCCAAGAC-3'
	Reverse: 5'-CTCCGGCGGAATAGAGAAAAG-3'
FBXO15	Forward: 5'- CCCACCAAACATAGACTCCGA-3'
	Reverse: 5'- CGAGCCTAATGTGCTCCTCT-3'
FBXO16	Forward: 5'- AGGATGGATTTGTAATCGCTGAC-3'
	Reverse: 5'- CGAAAAGCTGATAAAGGGGACT-3'
FBXO22	Forward: 5'-CGGAGCACCTTCGTGTTGA-3'
	Reverse: 5'- CACACACTCCCTCCATAAGCG-3'
FBXO41	Forward: 5'-CAGGCTGTCACGTACAGGAG-3'
	Reverse: 5'- AGGCAGCGGTTACTGAAGC-3'
FBXO47	Forward: 5'-AACTGGCAAGGCTCGTAGTC-3'

	Reverse: 5'- ACTGTCCAATCCATGCAGTACA-3'	
FBXO48	Forward: 5'- CAGGAGCTGGAATGACACAAT -3'	
	Reverse: 5'- TGGTAGGCTAATGGGAGAACAAA-3'	

Supplementary Table 3. Assay ID of Stealth siRNA for murine Fbxo proteins

Gene name	siRNA ID	Gene name	siRNA ID	Gene name	siRNA ID
Fbxo2	MSS279536	Fbxo15	MSS284755	Fbxo47	MSS223487
Fbxo7	MSS229880	Fbxo16	MSS224930	Fbxo48	MSS220187
Fbxo9	MSS245729	Fbxo22	MSS231239		
Fbxo10	MSS219088	Fbxo41	MSS221021		

Supplementary Table 4. Primers for murine Fbxo proteins

Gene name	Primer pairs
Fbxo2	Forward: 5'- CTGCTGTCGGAGAACGAAGAT-3'
	Reverse: 5'- GGCCCGTAGTCGATGAAGG-3'
Fbxo7	Forward: 5'- GTTGGGGTTCAGTTCTGATACC -3'
	Reverse: 5'- TCCTGGAGTGAGGAATGCTCT-3'
Fbxo9	Forward: 5'- TGGACCTCAGATCGTTAGAGC-3'
	Reverse: 5'- TGCCCCACACTTTCAAGCAA-3'
Fbxo10	Forward: 5'- AGCTGTGGCGTATGATCTTAGC-3'
	Reverse: 5'- GGTCTTAGAGGCAAGGTAGTGTT-3'
Fbxo15	Forward: 5'- CCTGGACAGAATGCCATCGG-3'
	Reverse: 5'- GCCAAATGATAAAAGCGTCTGC-3'
Fbxo16	Forward: 5'- GCACAGTCATTGATCTCCCTG-3'
	Reverse: 5'- GTTGCTGCTTCACGGTCTG-3'
Fbxo22	Forward: 5'- CAGCCTGGAAGAGTGTCGTG-3'
	Reverse: 5'- ACCTGATCCCATTGGAGTCAC-3'
Fbxo41	Forward: 5'- CGGCAGAATGCGAGAACATCA-3'
	Reverse: 5'- CTGAACCACCGTTGGTAGCC-3'
Fbxo47	Forward: 5'- GTGGCTGTTCAGTTCCTTTGC-3'
	Reverse: 5'- CCAACCTGCTGTTAAGGTCTGT-3'
Fbxo48	Forward: 5'- GCGGATGCTGAAAGGGGAAA-3'
	Reverse: 5'- TCTGAATGTCTAGCTGGCTGAA-3'
